# Heterologous Expression of the Marine-Derived Quorum Quenching Enzyme MomL Can Expand the Antibacterial Spectrum of *Bacillus brevis*

**DOI:** 10.3390/md17020128

**Published:** 2019-02-21

**Authors:** Jingjing Zhang, Jiayi Wang, Tao Feng, Rui Du, Xiaorong Tian, Yan Wang, Xiao-Hua Zhang

**Affiliations:** 1 MOE Key Laboratory of Marine Genetics and Breeding, College of Marine Life Sciences, Ocean University of China, Qingdao 266003, China; jingjingzhangnn@163.com (J.Z.); wangjiayi109911@163.com (J.W.); fengtao246@163.com (T.F.); drury612@163.com (R.D.); tianxr123@sina.com (X.T.); xhzhang@ouc.edu.cn (X.-H.Z.); 2 Laboratory for Marine Ecology and Environmental Science, Qingdao National Laboratory for Marine Science and Technology, Qingdao 266071, China; 3 Institute of Evolution & Marine Biodiversity, Ocean University of China, Qingdao 266003, China

**Keywords:** quorum sensing, quorum quenching enzyme, *N*-acylhomoserine lactones, *Bacillus brevis* expression system, biological control

## Abstract

Quorum sensing (QS) is closely associated with the production of multiple virulence factors in bacterial pathogens. *N*-acyl homoserine lactones (AHLs) are important QS signal molecules that modulate the virulence of gram-negative pathogenic bacteria. Enzymatic degradation of AHLs to interrupt QS, termed quorum quenching (QQ), has been considered a novel strategy for reduction of pathogenicity and prevention of bacterial disease. However, the low expression levels of QQ proteins in the original host bacteria has affected the applications of these proteins. Previously, we identified a novel marine QQ enzyme, named MomL, with high activity and promising biocontrol function. In this study, we linked the target fragment *momL* to pNCMO2, which provided a basis for the first heterologous expression of MomL in the antifungal and anti-gram-positive-bacteria biocontrol strain *Bacillus brevis*, and obtaining the recombinant strain named *Bb*MomL. The QQ activity of *Bb*MomL was confirmed using a series of bioassays. *Bb*MomL could not only degrade the exogenous signal molecule C6-HSL, but also the AHL signal molecules produced by the gram-negative pathogens *Pectobacterium carotovorum* subsp. *carotovorum* (*Pcc*) and *Pseudomonas aeruginosa* PAO1. In addition, *Bb*MomL significantly reduced the secretion of pathogenic factors and the pathogenicity of *Pcc* and *P. aeruginosa* PAO1. We tested the biocontrol function of *Bb*MomL for prevention of plant diseases in vitro. The result indicates that *Bb*MomL has a broad antibacterial spectrum. Compared with wild-type *B. brevis*, *Bb*MomL not only inhibited fungi and gram-positive bacterial pathogens but also considerably inhibited gram-negative bacterial pathogens. Moreover, the *Bacillus brevis* expression system has good application prospects and is an ideal host for expression and secretion of foreign proteins.

## 1. Introduction

**Figure 1:**
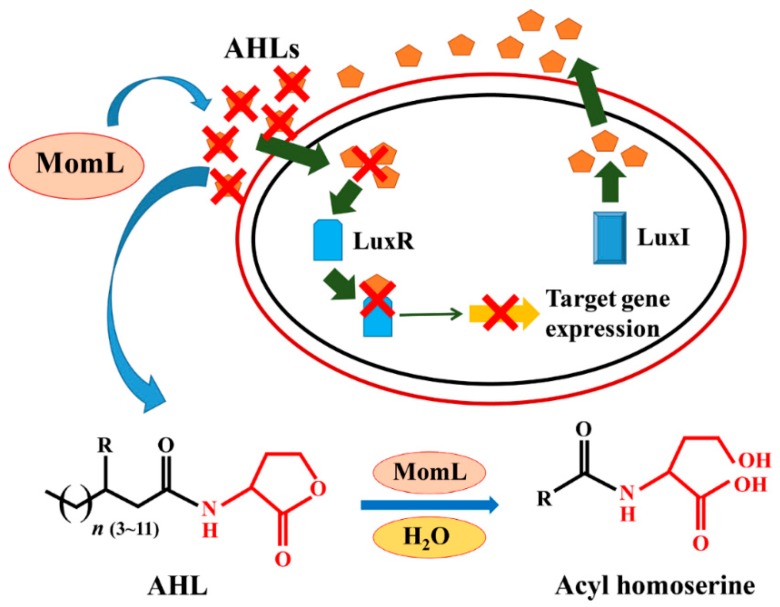
Mechanism of MomL-mediated degradation of *N*-acyl homoserine lactones (AHL) signal molecules.

Microbial quorum sensing (QS), also known as self-induction, is a self-sensing system in which microorganisms perceive population density by diffusion of secreted small molecules (self-inducers) between cells, and these small molecules regulate the expression of a series of genes [1]. The pathogenicity of many pathogens is mediated by the QS systems. For example, *Pectobacterium carotovorum* subsp. carotovorum (*Pcc*) can cause a soft rot of various plants, such as carrot and potato [2,3]; and *Pantoea stewartii* subsp. *stewartii* can cause symptoms of corn leaf blight [4]. Quorum quenching (QQ) is environmental protection, and a disease prevention strategy, that interferes with QS between microbial cells and blocks QS-dependent gene expression to prevent pathogenic infection [5]. *N*-acyl homoserine lactones (AHLs) are used by gram-negative pathogenic bacteria as autoinducers for intraspecies communications, and AHL lactonase can produce the corresponding acyl homoserine via cleavage of the lactone ring, thereby blocking the communication among bacteria [6]. MomL, which was identified from *Muricauda olearia* Th120, is a QQ enzyme with high AHL degradation activity [7]. This enzyme belongs to the metallo-β-lactamase family, in which QQ activity can be achieved via opening the lactone ring moiety (Figure 1) [8]. MomL shares 24.5% identity with AiiA, but the degradation efficiency of C6-HSL is approximately 10 times that of AiiA [9]. Previous research has shown that MomL significantly attenuated the virulence of *Pseudomonas aeruginosa* in a *Caenorhabditis elegans* infection model and has the potential for further development and application [7].

The selection of a suitable heterologous host, as well as efficient cloning, are key factors associated with successful heterologous expression [10]. Currently, the commonly used expression systems are the *Escherichia coli* expression system, the *Bacillus subtilis* expression system and the yeast expression system. In this paper, we use a prokaryotic expression system, namely, the *Bacillus brevis* expression system [11,12,13,14]. The *B. brevis* expression system, characterized by the high-efficiency secretory expression, offers excellent protein production characteristics, allowing the production of a large number of heterologous proteins. Moreover, *B. brevis* can secrete a variety of active substances, such as chitinase and gramicidin, which have strong inhibitory effects on many pathogenic fungi and especially on gram-positive bacteria, but the effect on gram-negative bacteria was weak [15,16]. Tostadin, a novel small antibacterial peptide, was obtained from the liquid culture of *Brevibacillus brevis* XDH, which is a broad-spectrum antagonistic bacterium. The study showed that this peptide had a strong inhibitory effect on many pathogens both in vivo and in vitro [15]. Gramicidin S and polymyxins are small cationic cyclic peptides that act as effective antibiotics against pathogenic bacteria by disrupting the integrity of bacterial membranes [16]. AiiA, the first identified AHL lactonase with AHL degradation activity, has been identified in several strains of *Bacillus* species [17,18]. Previous reports have suggested that the AiiA in gram-positive bacteria plays a greater role in detoxification than in quenching [19]. *Bacillus* sp. do not produce AHL signal molecules. In theory, the heterologous expression of the AHL lactonase MomL in *B. brevis* does not affect intraspecific information exchange in this organism. Examples of protein production using this system are listed in Table 1. High expression levels have been achieved for a variety of proteins (enzymes, antigens and cytokines) regardless of gene origin (bacteria, archaea and eukaryotes).

In this study, we selected pNCMO2 and *B. brevis* as the shuttle expression vector and the heterologous host cell, respectively, to achieve highly efficient expression of the marine-derived QQ enzyme MomL. The P2 promoter, derived from a cell wall protein of the host bacterium, was used as the promoter for pNCMO2 expression. The virulence factors of the plant pathogen *Pcc* are controlled by cell density-dependent regulation, and the QQ enzymes are expected to provide a new tool for attenuation of the development of soft rot in plants. By conduction signal molecule degradation experiments and in vitro detection of the control of plant soft rot by the recombinant strain *Bb*MomL, we found that the recombinant strain *Bb*MomL can effectively degrade the AHL molecules produced by pathogenic bacteria, block the QS system and reduce the pathogenicity of the bacteria. Our results provide a biological strategy for disruption of bacterial QS and further expand the potential applications of *B. brevis* in biological control.

## 2. Results

### 2.1. Construction of the Recombinant Expression Strain BbMomL and Detection of AHL Degradation Activity

**Figure 2:**
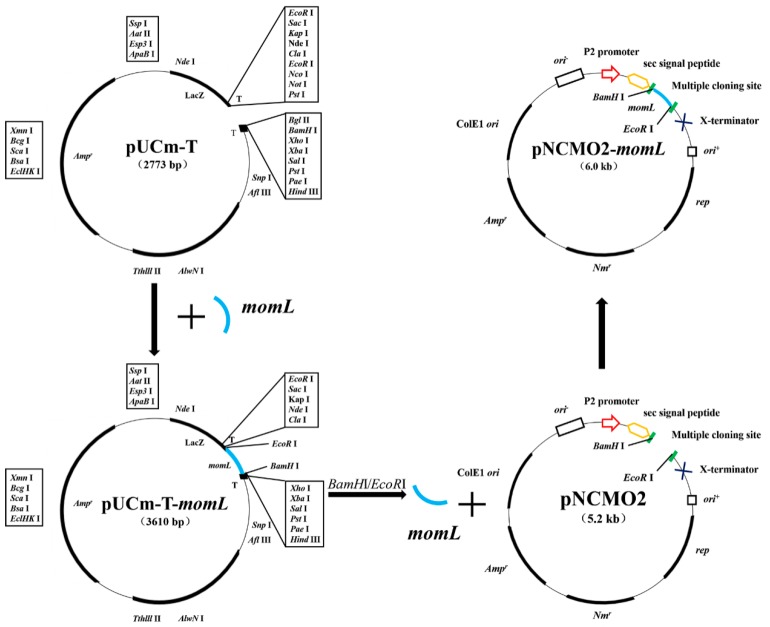
Flow chart of the construction of the gene expression vector pNCMO2-*momL*.

To improve the transformation efficiency and increase the possibility of successful transformation, we prepared the recombinant plasmid pUC-T-*momL* and transformed it into *E. coli* JM109. Then, we constructed the expression plasmid pNCMO2-*momL* using pNCMO2 as a vector, and the constructed recombinant plasmid contained an 837-bp DNA fragment of encoding the marine QQ enzyme MomL (Figure 2).

**Figure 3:**
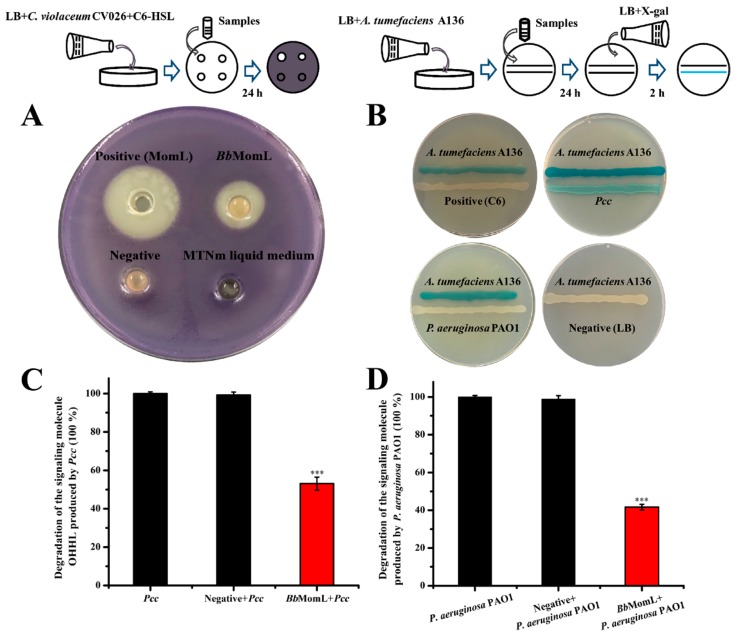
Detection of the quorum quenching (QQ) activity of *Bb*MomL. (**A**) Degradation of the exogenous signal molecule C6-HSL by *Bb*MomL. The concentration of the pure MomL protein was 1.363 mg/mL. (**B**) Determination of whether *Pcc* and *P. aeruginosa* PAO1 can generate AHL signal molecules. (**C**) Degradation of the 3-oxo-hexanoyl-homoserine-lactone (OHHL) signal molecule by *Bb*MomL. (**D**) Degradation of the signal molecule produced by *P. aeruginosa* PAO1 by *Bb*MomL. The negative control was *B. brevis* (pNCMO2 transformant). The results shown are representative of biological duplicates. Error bars represent the standard deviations of three replicates. A *t*-test of unpaired unequal variance was performed for testing differences between groups. For statistical analysis, ***, **, and * indicate *P* < 0.001, *P* < 0.01, and *P* < 0.05, respectively.

In this experiment, *Chromobacterium violaceum* CV026 and *Agrobacterium tumefaciens* A136 were used as indicator strains [32,33]. The C6-HSL, a kind of AHL signal molecule, is one of the most widely studied QS molecules. In the presence of C6-HSL, *C. violaceum* CV026 is capable of producing violacein, and when QQ enzymes are added, the production of violacein is inhibited. Specifically, *C. violaceum* CV026 did not produce violacein when the purified protein MomL or *Bb*MomL was added, indicating that the recombinant strain *Bb*MomL exhibits QQ activity and degrades exogenous C6-HSL (Figure 3A). The concentration of purified MomL was higher than the supernatant of *Bb*MomL, therefore, the QQ activity of *Bb*MomL was significantly lower than the pure MomL protein on the indicator plate. The indicator strain *A. tumefaciens* A136 expresses β-galactosidase and decomposes X-gal under the induction of AHL signaling molecule to produce a blue substrate. In this study, we found that *A. tumefaciens* A136 turned into blue under the C6-HSL, *Pcc* or *P. aeruginosa* PAO1, while the negative control did not show this phenomenon (Figure 3B). The result showed that both *Pcc* and *P. aeruginosa* PAO1 could produce AHL signaling molecules, and *Bb*MomL had significant degradation effect on them. The results further confirmed that the marine-derived QQ enzyme MomL could be expressed in *B. brevis* and confer a new property to this bacterium, namely, the ability to degrade AHL signal molecules (Figure 3C,D).

### 2.2. Effects of BbMomL on the Growth of Pcc and P. aeruginosa PAO1

**Figure 4:**
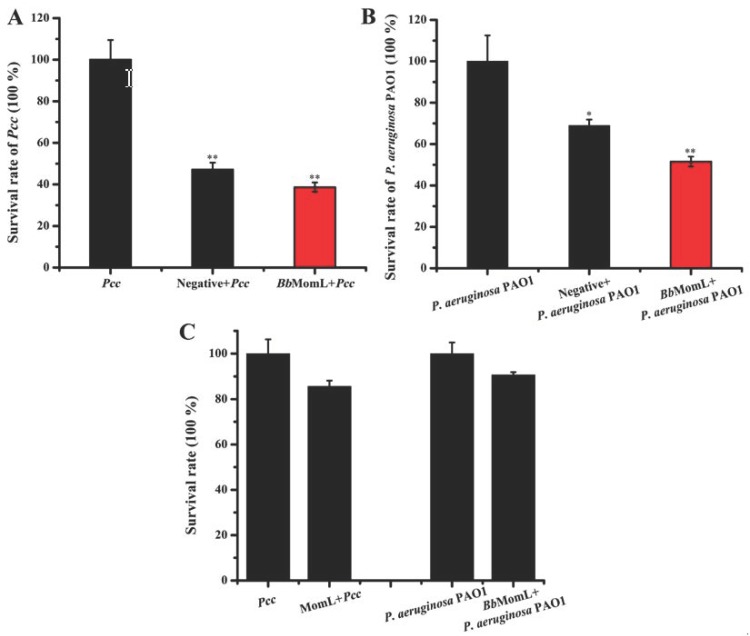
Effects of *Bb*MomL on the survival rates of *Pcc* and *P. aeruginosa* PAO1. (**A**) Effect of *Bb*MomL on the survival rate of *Pcc*. (**B**) Effect of *Bb*MomL on the survival rate of *P. aeruginosa* PAO1. (**C**) Effects of the recombinant MomL protein on the survival rates of *Pcc* and *P. aeruginosa* PAO1. The concentration of the pure MomL protein was 1.363 mg/mL. The negative control was *B. brevis* (pNCMO2 transformant). The results shown are representative of biological duplicates. Error bars represent the standard deviations of three replicates. A *t*-test of unpaired unequal variance was performed for testing differences between groups. For statistical analysis, ** and * indicate *P* < 0.01 and *P* < 0.05, respectively.

We determined the impact of MomL on the survival rate of *Pcc* and *P. aeruginosa* PAO1 using the dilution plate count method. The results showed that the survival rate of *Pcc* and *P. aeruginosa* PAO1 decreased significantly after coculture with *Bb*MomL and *B. brevis* (pNCMO2 transformant), respectively (Figure 4A,B). However, it is not clear whether the decrease of the survival rate is caused by the expression of the *momL* gene. Therefore, subsequent supplementary experiments were carried out. We found that the survival rates of *Pcc* and *P. aeruginosa* PAO1 after coculture with pure MomL protein were not significantly reduced (Figure 4C). This finding indicates that the QQ enzyme MomL does not directly inhibit bacterial growth and does not exert a selection pressure on bacterial survival.

### 2.3. Isolation of the BbMomL Extracellular Protein and Analysis of AHL Degradation Activity

**Figure 5:**
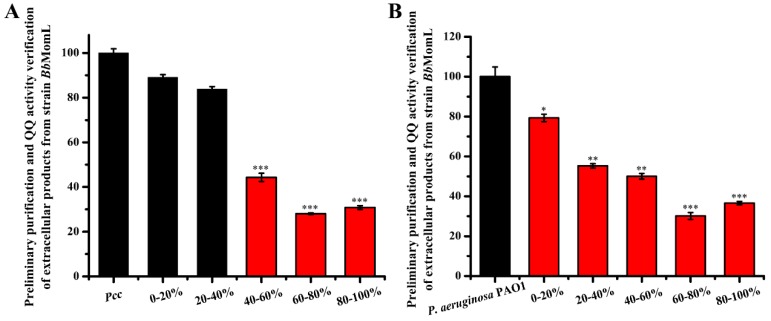
Effects of different components of the *Bb*MomL extracellular protein on the signal molecules produced by *Pcc* and *P. aeruginosa* PAO1. (**A**) Effects of different components of *Bb*MomL extracellular protein on signal molecules produced by *Pcc*. (**B**) Effects of different components of the *Bb*MomL extracellular protein on signal molecules produced by *P. aeruginosa* PAO1. The X-axis refers to different protein components isolated from 0–20%, 20–40%, 40–60%, 60–80%, and 80–100% ammonium sulfate. The Y-axis refers to the QQ activity of different protein components separated from the culture supernatant. The results shown are representative of biological duplicates. Error bars represent the standard deviations of three replicates. A *t*-test of unpaired unequal variance was performed for testing differences between groups. For statistical analysis, ***, ** and * indicate *P* < 0.001, *P* < 0.01, and *P* < 0.05, respectively.

*Bacillus brevis*, as a promising heterologous expression system, can achieve extracellular expression of MomL. In this study, the extracellular protein was isolated by the ammonium sulfate step-precipitation method, and the *A. tumefaciens* A136 plate-based method was used to detect the QQ activity of different components. The components with degradation activity toward the signal molecules produced by *Pcc* were concentrated mainly in the ammonium sulfate concentration (*w*/*v*) ranges of 40–60%, 60–80% and 80–100%, and the QQ activity reached up to 50%, while the other components did not exhibit QQ activity (Figure 5A).

Upon verification of the degradation of the signal molecules produced by *P. aeruginosa* PAO1 by different protein components, we found that the degradation efficiency of the 0–20% ammonium sulfate component toward the AHL signal molecule was only 20%. The degradation efficiencies of the 20–40% and 40–60% ammonium sulfate components reached up to 40%, and the highest degradation efficiencies were observed for the 60–80% and 80–100% ammonium sulfate components, reached 60% (Figure 5B). The above results indicated that the *Bb*MomL extracellular protein was separated into different components, which exhibited different degradation efficiencies toward the signal molecules produced by *Pcc* and *P. aeruginosa* PAO1.

### 2.4. In Vitro Experiments to Evaluate the Ability of BbMomL to Inhibit the Virulence Factors of Pathogenic Bacteria

**Figure 6:**
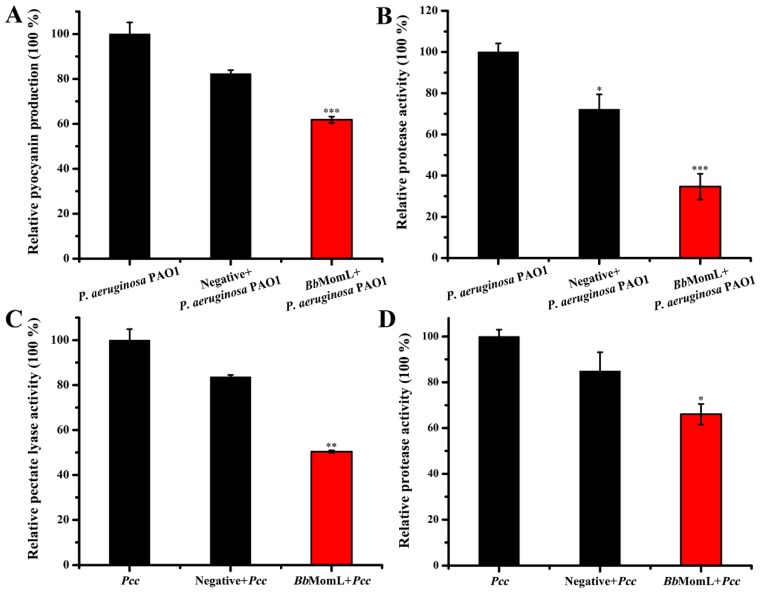
Inhibitory effect of *Bb*MomL on pathogenic factors produced by *Pcc* and *P. aeruginosa* PAO1. (**A**) Relative production of pyocyanin of *P. aeruginosa* PAO1. (**B**) Relative protease activity of *P. aeruginosa* PAO1. (**C**) Relative pectate lyase activity of *Pcc*. (**D**) Relative protease activity of *Pcc*. The negative control was *B. brevis* (pNCMO2 transformant). The results shown are representative of biological duplicates. Error bars represent the standard deviations of three replicates. A *t*-test of unpaired unequal variance was performed for testing differences between groups. For statistical analysis, ***, ** and * indicate *P* < 0.001, *P* < 0.01, and *P* < 0.05, respectively.

The inhibitory effect of *Bb*MomL on the virulence factors of pathogens was detected. We found that after coculture with the recombinant strain *Bb*MomL, the secretion of pyocyanin and extracellular protease of *P. aeruginosa* PAO1 and extracellular proteases and pectate lyase of *Pcc* was significantly reduced. The recombinant strain *Bb*MomL could significantly reduce the secretion of pathogenic factors of *P. aeruginosa* PAO1 and *Pcc*, so we suggested that it could reduce the pathogenicity (Figure 6). At the same time, we found that the negative control also slightly inhibited the virulence factors, which may be due to some unidentified active substances produced by *B. brevi*.

### 2.5. Analysis of Growth and Protein Content of BbMomL in Different Media and Secretion of the Target Protein at Different Times

**Figure 7:**
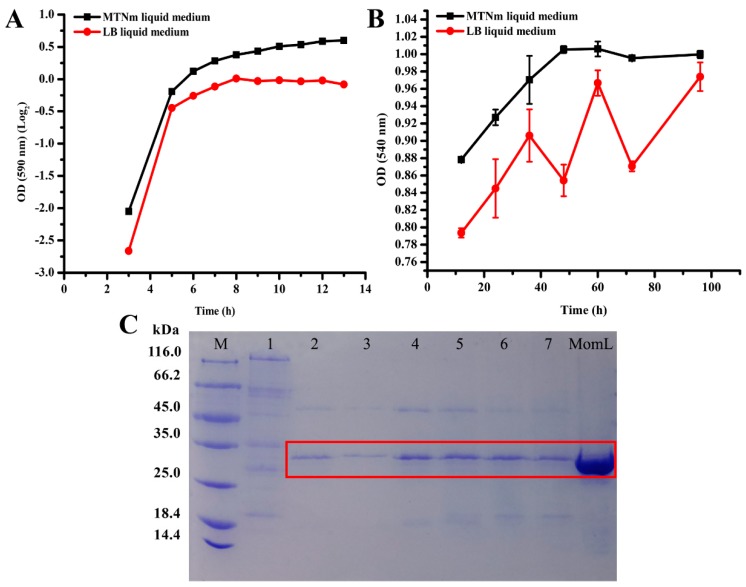
Analysis of the growth and protein content of *Bb*MomL in different media and by sodium dodecyl sulfate-polyacrylamide gel electrophoresis (SDS-PAGE). (**A**) Analysis of *Bb*MomL growth in LB and MTNm. (**B**) Analysis of protein secretion by *Bb*MomL in LB and MTNm. (**C**) The SDS-PAGE analysis of secretion of the target protein MomL at different culture time points. M: Marker; 1: Negative control; 2: 12 h; 3: 24 h; 4: 48 h; 5: 60 h; 6: 84 h; 7: 96 h; MomL: Positive control. The results shown are representative of biological duplicates. Error bars represent the standard deviations of three replicates.

It has been reported that *B. brevis* can grow in different media. In this experiment, we compared the growth and protein secretion activity of *Bb*MomL in two different media, namely Luria-Bertani (LB) and MTNm media. The growth rate of *Bb*MomL in MTNm medium was higher than that in LB medium (Figure 7A). Moreover, the protein secretion activity of *Bb*MomL in MTNm liquid medium was higher and more stable than that in LB (Figure 7B). To verify the change in the secretion of the target protein MomL at different time points in MTNm medium, we performed sodium dodecyl sulfate-polyacrylamide gel electrophoresis (SDS-PAGE). The SDS-PAGE analysis showed that the intensity of the protein bands increased with culture time, indicating that the level of the target protein MomL in the culture supernatant also increased with incubation time (Figure 7C).

### 2.6. Inhibitory Effect of BbMomL on Bacterial Soft Rot of Plants

**Figure 8:**
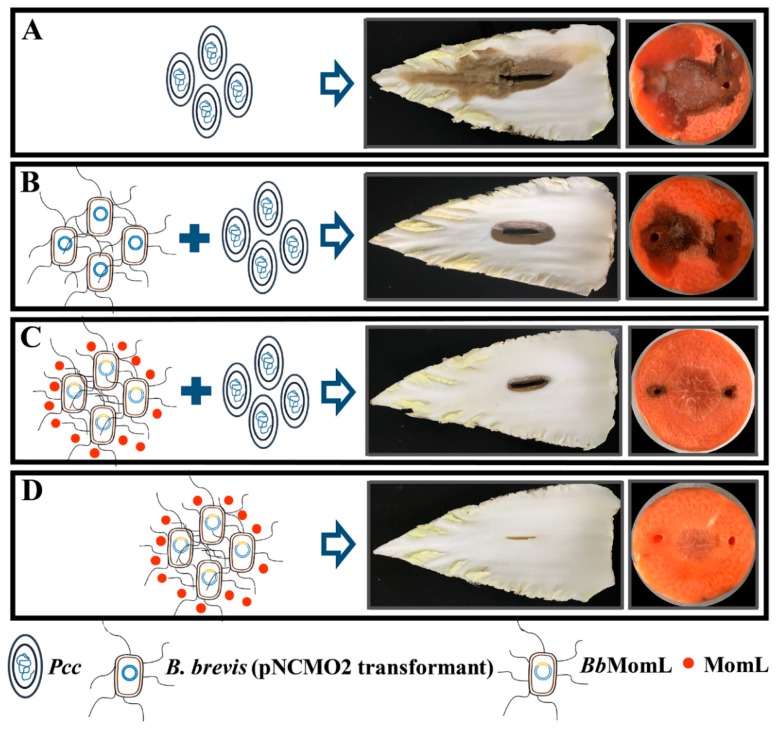
In vitro control efficacy of *Bb*MomL against *Pcc* soft rot of Chinese cabbage and carrot root tissues. (**A**) *Pcc*; (**B**) *Pcc* with *B. brevis* (pNCMO2 transformant); (**C**) *Pcc* with the *Bb*MomL strain; (**D**) the *Bb*MomL strain.

*Pcc* is one of the main pathogens that cause plant bacterial soft rot, affecting various vegetables, cash crops and ornamental plants. To verify the application potential of *Bb*MomL to treat plant soft rot caused by *Pcc*, we infected root tissues of Chinese cabbage and carrot *in vitro*. Inoculation of Chinese cabbage and carrot root tissues with the pathogenic bacteria *Pcc* alone caused obvious symptoms of bacterial soft rot. The rotten area of the plant tissue was significantly reduced, and the symptoms of bacterial soft rot in the plants were improved after coinoculation with *Pcc* and the recombinant *Bb*MomL strain, while symptoms of tissue decay were only slightly improved by coinoculation with *Pcc* and a negative control (Figure 8). This phenomenon may be due to the antagonistic effect between *Pcc* and *B. brevis* (pNCMO2 transformant). According to the results, we speculated that one possible reason why *Bb*MomL inhibited the soft rot in Chinese cabbage and carrot was that *Bb*MomL inhibited the production of pectate lyase and protease in *Pcc*. In addition, when only *Bb*MomL was used to infect Chinese cabbage and carrot tissues, there were no soft rot symptoms in the root tissues, indicating that *Bb*MomL had no toxic effects on the plant tissues.

## 3. Discussion

Quorum sensing (QS) is a population-dependent behavior that enables bacteria to sense and communicate with other neighbors and then to regulate multiple genes in response to external environmental changes [1,34,35,36,37,38,39]. Among them, QS induced by AHLs (QSA) was identified in multiple bacterial species, most of which were common pathogens existing in various environments. Previous studies have shown that QSA is closely related to the pathogenicity, virulence factor production and biofilm formation in multiple pathogens, including many antibiotic-resistant microorganisms [40,41,42,43,44,45]. Traditionally, antibiotic therapy is recognized as an effective way for the control of bacterial pathogens. However, the overdose of antibiotics has accelerated the emergence of multidrug-resistant bacteria [46,47,48]. The dilemma has prompted the research of novel antibacterial strategies. Interfering with QSA systems via quorum quenching (QQ) represents a promising strategy for the treatment of bacterial diseases [5]. Theoretically, QQ could decrease the expression of virulence factors produced by any pathogens under the control of QSA. Many QQ agents have been discovered, and their mechanism of action has also been well studied [7,49,50,51]. However, it is largely unknown about how to efficiently utilize these QQ agents and express them in ideal microorganism hosts.

**Figure 9:**
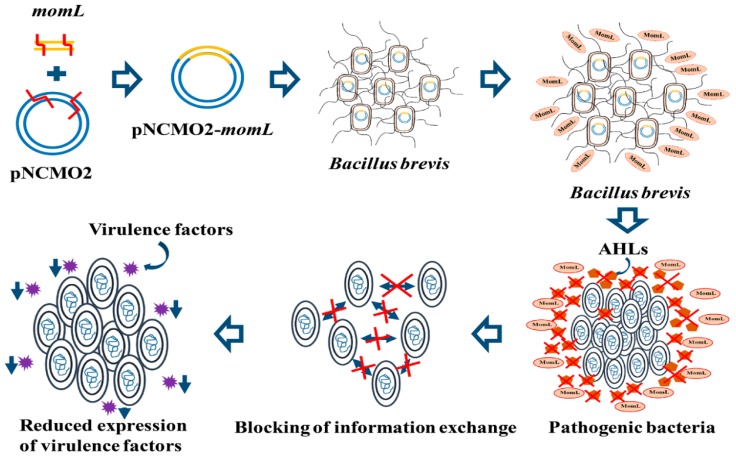
Schematic diagram of the mechanism of action of MomL.

In this study, we carried out heterologous expression of the marine-derived QQ agent MomL in *B. brevis*, which is an antifungal and anti-gram-positive-bacteria biocontrol bacterial microorganism, and obtained the recombinant strain named *Bb*MomL. This is novel that *B. brevis* was used as a heterologous expression vector for marine QQ enzyme. Moreover, the study of QQ agents’ heterologous expression is at the initial stage, and this is the first time MomL was expressed in *Bacillus genus*. Our result showed that *Bb*MomL could degrade both the exogenous signal molecule C6-HSL and the AHLs produced by the gram-negative pathogens *Pcc* and *P. aeruginosa* PAO1. In addition, *Bb*MomL significantly reduced the production of pathogenic factors and the pathogenicity of *Pcc* and *P. aeruginosa* PAO1. The reason we chose *Pcc* and *P. aeruginosa* PAO1 as the study materials was that these two kinds of bacteria are widely existed and have serious pathogenicity to animals and crops respectively. More importantly, previous studies have shown that the production of virulence factors of these two pathogens are closely related to AHL-mediated quorum sensing process. The heterologous expression of MomL in *B. brevis* can lead to the production of MomL with biological activity. MomL can degrade AHL signal molecules produced by pathogenic bacteria and interrupt the communication among pathogenic bacteria. Therefore, it has an obvious biological control effect (Figure 9). Besides, our result indicated that *Bb*MomL had a broad antibacterial spectrum. *Bb*MomL not only inhibited fungi and gram-positive bacterial pathogens, but also considerably inhibited gram-negative bacterial pathogens. This result raises our thinking that if we express MomL or other QQ agents in other beneficial bacteria, will they also improve the antibacterial activity of the bacteria? The mechanism that MomL enhanced the antimicrobial ability of beneficial bacteria is an interesting scientific question deserved further study. In the study, we also found an interesting phenomenon that *Bb*MomL has a significant inhibitory effect on plant soft rot caused by *Pcc*, while *B. brevis* also showed a slight therapeutic effect. We speculated that the reason for this phenomenon was some unidentified active substances secreted by *B. brevis* had a certain therapeutic effect on soft rot, meanwhile, they also had a certain effected on the survival of pathogenic bacteria. In addition, we found that MomL could reduce the survival rate of *Pcc* and *P. aeruginosa* PAO1, which was not consistent with the theory that MomL cannot directly kill bacteria. We speculated that MomL indirectly affected the survival rate of the pathogenic bacteria. MomL can cause pathogenic bacteria to lose their survival advantage in the presence of other alien bacteria. Several studies have shown that AHL-mediated QS affect a series of physiological behaviors of bacteria, such as the production of pathogenic factors, biofilm formation, antibiotic resistance and stress resistance. MomL resulted in the loss of AHL and then these physiological behaviors were affected, which in turn affected the viability of bacteria.

The heterologous expression has received increased attention because of the low expression of target proteins in the original host bacteria. Now there are a variety of heterologous expression systems, which allows us to select the most appropriate one for different target proteins. A series of heterologous expression systems have their own advantages and disadvantages features. For example, although the *E. coli* expression system is easy to operate, the expressed products readily form inclusion bodies and lose biological activity. Meanwhile, the extracellular expression of protease may hydrolyze the proteins of the host bacteria and cause host bacterial death [11]. In recent years, there have been many successful examples of studies on the secretion and expression of foreign proteins in *Bacillus*. A series of effective expression systems have been established, such as *B. subtilis* and *B. brevis* expression systems [52]. Currently, the *B. subtilis* expression system is among the most important prokaryotic expression systems, following *E. coli*, and has been successfully used a host species in industrial applications [12,53,54,55]. However, as an expression host, *B. subtilis* also exhibits some limitations that restrict the application of this species in industrial production, such as high extracellular protease activity and high degradation susceptibility of the expression products [53]. Compared with *B. subtilis*, the *B. brevis* expression system has not only a strong ability to secrete proteins, but also low extracellular protease activity. For example, the extracellular protease activity level of *B. brevis* 47 is only 1.6% that of *B. subtilis*, and the protease activity of *B. brevis* HPD31 is almost undetectable [56,57]. In addition, disulfide bond oxidoreductase (Dsb) and peptide proline cis-trans isomerase (PPIase), which can promote the correct folding of polypeptides, have also been detected in *B. brevis* culture supernatant [58]. Therefore, *B. brevis* has the natural advantage of being able to secrete and express exogenous proteins. There have been successful reports on the use of *B. brevis* for construction of a secretion system for foreign proteins. The production of a mouse/human chimeric antibody against human prourokinase by *B. brevis* reached 100 mg/L by a simple shake flask culture method [59]. Peng et al. [60] also successfully expressed α-amylase in *B. brevis*. These results all show the considerable application potential of *B. brevis* in the secretion and expression of exogenous proteins.

## 4. Materials and Methods

### 4.1. Strains, Plasmids and Culture Conditions

The bacterial strains and plasmids used in this study are described in Table S1. *C. violaceum* CV026 and *A. tumefaciens* A136 were used as indicator strains in the AHL activity bioassay. *M.* Th120, *C. violaceum* CV026, *Pcc* and *P. aeruginosa* PAO1 were routinely grown on LB agar medium at 28 °C. When the effects of *Bb*MomL on in vitro pyocyanin production in *P. aeruginosa* PAO1 were determined, the cells were cultured in *Pseudomonas* broth (PB medium) [61]. The recombinant strain *Bb*MomL was cultured in MTNm liquid medium at 37 °C [62]. The media components are described in Table S2. When required, antibiotics were used at the following concentrations: 10 μg/mL neomycin for *Bb*MomL; and 4.5 μg/mL tetracycline and 50 μg/mL spectamycin for *A. tumefaciens* A136. C6-HSL was purchased from Sigma-Aldrich (St. Louis, MO, USA) and prepared in dimethyl sulfoxide (DMSO). Genomic DNA of *M. olearia* Th120 was extracted by the phenol-chloroform method. Plasmids and DNA fragment extraction were performed following the instructions included with the kits purchased from OMEGA (Plasmid Mini Kit I and Gel Extraction Kit, Omega Bio-Tek, Norcross, GA, USA). The *Bacillus brevis* expression system (cat. #HB200), PrimeSTAR GXL DNA polymerase (Code No. R050A), Solution I (cat. #6022-1) and restriction enzymes were purchased from TaKaRa (TaKaRa Bio Group, Shiga, Japan). PCR primers were synthesized by Tsingke Biological Technology Company (Qingdao, China).

### 4.2. Plasmids Construction

The *B. brevis* expression system was used for the expression of native MomL according to the manufacturer’s instructions. To improve the transformation efficiency, we linked the target gene *momL* with pUC-T vector and transformed it into *E. coli* JM109. Insertion of the lac operators into pNCMO2 can weaken the activity of promoters in *E. coli*, and thus makes it necessary to use the *E. coli* JM109 host which F factor (*lacIq*) has been integrated. Briefly, an 837-bp DNA fragment encoding MomL was obtained using *M. olearia* Th120 genomic DNA as a template. Based on the *momL* sequence, a His-tag was included at the C-terminus. Polymerase chain reaction (PCR) was carried out using specific primers (forward primer, 5′-CGCGGATCCAAAAAGGAAGCT-3′; reverse primer, 5′-CCGGAATTCGTGGTGGTGGTG-3′) with BamH I and EcoR I restriction sites for directional cloning of MomL. PCR was conducted as follows: Denaturation at 95 °C for 2 min followed by 30 cycles of 95 °C for 10 s, 60 °C for 15 s and 68 °C for 1 min and an elongation step of 72 °C for 2 min (PrimeSTAR GXL DNA polymerase can amplify 1 kb every 10 s, so the extension time was set according to the length of the target fragment). The amplified DNA was purified, digested with BamH I and EcoR I, and cloned into the pUCm-T vector (previously digested with the same enzymes) by Solution I to construct the recombinant pUCm-T-*momL* plasmid. This plasmid was then transformed into *E. coli* JM109 cells. The positive clones were screened by blue-white selection on LB plates containing 50 μg/mL ampicillin and then sequenced to confirm the identity of the amplicon. The pUCm-T-*momL* plasmid was extracted with kits, digested with the restriction endonuclease BamH I and EcoR I, and cloned into the pNCMO2 vector (previously digested with the same enzymes) with Solution I to construct the recombinant pNCMO2-*momL* [62]. This construct was transformed into *E. coli* JM109 cells, grown overnight at 37 °C in plates with LB agar medium containing ampicillin-neomycin (10 μg/mL), and colonies were screened for the presence of the pNCMO2-*momL* construct. Next, the correct positive transformant plasmid was extracted, and the target plasmid was introduced into *B. brevis* competent cells according to the manufacturer’s instructions and grown overnight at 37 °C in MTNm plates containing neomycin (10 μg/mL) to screen the positive transformants and obtain *Bb*MomL.

### 4.3. Bioassay for AHL Degradation Activity

The AHL degradation activity of *Bb*MomL was detected using the strain *C. violaceum* CV026 according to the method described by McClean [32]. Detection of QQ activity was performed based on the inhibition of violacein production by the *C. violaceum* CV026 strain in culture medium supplemented with an exogenous QS signal molecule, namely, C6-HSL [63]. Fifteen milliliters of molten semisolid LB agar (1%, *w*/*v*) was seeded with 1 mL of an overnight LB culture of *C. violaceum* CV026. Then, 7.5 µL of C6-HSL (DMSO, 1 mM) was added before the agar was poured over the plates. When the agar solidified in the screening plates, the solidified semisolid medium was perforated using an Oxford cup. Then, 150 μL of the culture was added to the well, and 10 μL of the purified recombinant MomL protein (1.363 mg/mL) was used as the positive control. The plates were incubated overnight at 28 °C.

To determine whether *Pcc* and *P. aeruginosa* PAO1 can produce AHL signal molecules, we performed experiments using the indicator strain *A. tumefaciens* A136. The *A. tumefaciens* A136 and test strains were streaked on an LB plate in the form of a “=” symbol, and the plate was incubated at 28 °C for 12 h. Then, X-gal (100 μg/mL) was added to LB semisolid medium (1% agar), mixed and poured into the above plate, which was then covered and incubated at 28 °C for 2 h.

We detected the degradation of AHL signal molecules produced by *Pcc* and *P. aeruginosa* PAO1 by *Bb*MomL using the methods described above. The bacterial supernatants of *Bb*MomL coculture with *Pcc* and *P. aeruginosa* PAO1 were separately placed in the plate containing the strain *C. violaceum* CV026, and cultured overnight at 28 °C to observe the results.

### 4.4. Effects of BbMomL on the Survival of Pcc and P. aeruginosa PAO1

*Pcc* or *P. aeruginosa* PAO1 and *Bb*MomL were separately cultured to the exponential phase and mixed at a ratio of 1:1. The cultures were cultivated on a shaker (170 rpm) at 28 °C for 8 h. The bacterial suspensions were diluted with fresh LB and plated on LB agar. After 15 h of incubation at 28 °C, colonies were counted. The number of colonies on the *Pcc* and *P. aeruginosa* PAO1 plates were set to 100%. Each experiment involved three repetitions.

### 4.5. Isolation and Purification of Recombinant Enzymes and AHL Bioassay of Pcc and P. aeruginosa PAO1

The extracellular protein of *Bb*MomL was isolated and purified by the ammonium sulfate step-precipitation. Based on the volume of the extracellular product, ammonium sulfate was added to achieve a saturation of 20%. When the ammonium sulfate was completely dissolved, the extracellular product was centrifuged at 12,000× *g* for 30 min at 4 °C to obtain a protein precipitate. The above steps were repeated, and ammonium sulfate was sequentially added to the supernatant to achieve saturation levels of 40%, 60%, 80% and 100%, and the protein precipitates were collected by centrifugation. The protein precipitates were dissolved in PBS and dialyzed for 24 h in the same solution. Using strain *A. tumefaciens* A136 as an indicator strain, the QQ activity of the extracellular protein was detected by the Oxford cup method.

### 4.6. Effects of BbMomL on Virulence Factor Production in Pcc and P. aeruginosa PAO1

The inhibitory effect of *Bb*MomL on the synthesis of pyocyanin in *P. aeruginosa* PAO1 was measured as described by Essar [64]. The total volume of each experimental group was 5 mL. *Bb*MomL and *P. aeruginosa* PAO1 with the same cell density were mixed at a ratio of 1:1 and cultivated at 170 rpm for 24 h at 28 °C. One milliliter of the culture solution was removed, and 800 μL of chloroform was added for extraction. After centrifugation at 13,000× *g* for 2 min, 500 μL was extracted from the lower chloroform layer and uniformly mixed with 1.5 mL of 200 mM hydrochloric acid, and then, the OD520 was measured. Each experiment involved three repetitions.

The effect of *Bb*MomL on the extracellular protease activity of *Pcc* and *P. aeruginosa* PAO1 was determined according to the method described by Hentzer [65,66]. The coculture samples were incubated at 28 °C for 24 h, and the OD590 was measured. After centrifugation at 13,000× *g* for 10 min at 4 °C, 100 μL of the supernatant was mixed with a 50 mM Tris/HCl solution (pH = 7.8) containing 2% azocasein and incubated at 37 °C for 2 h. The reaction was stopped by addition of 200 μL of 10% trichloroacetic acid solution, allowed to stand for 2 min, and centrifuged at 13,000× *g* for 5 min. A total of 100 μL of the supernatant was removed and mixed with 100 μL of 525 mM sodium hydroxide solution, and then, 200 μL of the mixture was placed in a 96-well plate. The OD415 was measured, and the protease activity of each sample was calculated according to the formula OD415/(OD590 × 100 μL).

This experiment was performed to determine the inhibitory effect of *Bb*MomL on pectate lyase in *Pcc*. The absorbance at 590 nm of the mixtures was determined. Then 600 μL of the coculture supernatant, 100 μL of CaCl_2_ solution and 800 μL of glycine-sodium hydroxide buffer (50 mM, pH = 9.5) containing 0.2% (*w*/*v*) polygalacturonic acid were uniformly mixed. The reaction was stopped by the addition of 500 μL of HCl (50 mM) after 5 min. The OD235 was measured, and the relative activity of pectate lyase was determined according to the formula OD235/OD590.

### 4.7. Comparison of the Growth and Protein Content of BbMomL in Different Media and by SDS-PAGE Analysis

*Bb*MomL was cultured separately in LB and MTNm liquid medium containing 10 μg/mL neomycin at 37 °C with shaking at 170 rpm. The absorbance values at 590 nm was determined at different time points, and a curve was generated. The concentration of the total proteins was determined by the Bradford method in this study [67]. The Bradford assay relies on the binding of the dye Coomassie Blue G250 to protein in cell-free supernatants. The cell-free culture supernatants at different time points were mixed with the dye Coomassie Blue G250 solution and its absorbance at 540 nm was determined. Three parallel sample sets were examined for each set of experiments. The supernatants of the recombinant *Bb*MomL cultured in MTNm medium were collected by centrifugation, and equal amounts of total protein were loaded onto SDS-PAGE gels to detect the secretion of the target protein MomL at different time points [68].

### 4.8. Inhibition of Plant Bacterial Soft Rot

To examine the biocontrol effect of *Bb*MomL on soft rot disease caused by *Pcc*, an in vitro disease control efficacy test was performed using Chinese cabbage and carrot roots. Coculture suspensions of *Bb*MomL with *Pcc* were prepared as previously described. Washed Chinese cabbage leaves were disinfected with 70% ethanol for 30 s and dried on a clean bench. A 2 cm long wound was made with a sterile razor in the center of the leaves, and 30 μL of the mixed bacterial suspension was applied to the wound. A *Pcc* bacterial suspension was used as a positive control. The compound-treated Chinese cabbage leaves were incubated for 48 h at 28 °C in a plastic bag with filter paper moistened with 5 mL of sterile water to prevent excessive drying, and then, the lesions were examined. This experiment was conducted three times independently with three replicates in each trial. The infection experiment with carrot root tissues was performed as described previously with slight modifications [63,69]. The surface-sterilized carrot roots were cut into 0.7 cm thick sliced, and two wells (5 mm diameter) were made in each slice. Then 30 μL of the coculture suspension was applied to the wells, while the control group was inoculated with only pathogenic or antagonistic bacterial suspension. The compound-treated carrot slices were placed on Petri dishes with wet filter paper to prevent excessive drying and incubated for 48 h at 28 °C, and then, the lesions were examined. This experiment was conducted three times independently, with three replicates in each trial.

## Figures and Tables

**Table 1:** Examples of heterologous protein expression with the *Bacillus brevis* expression system.

Protein	Origin	Quantity of Expression (g/L)	References
**Enzymes**			
Alpha-Amylase	*B. licheniformis*	3.7	[20]
Sphingomyelinase	*B. cereus*	3.0	[21]
Xylanase	*B. halodurans*	0.2	[21]
CGTase	*B. macerans*	1.5	[22]
Chitosanase	*B. circulans*	1.4	[23]
Hyperthermophilic protease	*A. pernix*	0.1	[24]
Hyperthermophilic nuclease	*P. horikoshii*	0.7	[25]
PDI	Human	1.0	[26]
**Antigens**			
Surface antigen	*E. rhusiopathiae*	0.9	[21]
Surface antigen	*T. pallidum*	0.8	[21]
**Cytokines**			
EGF	Human	1.5	[27]
IL-2	Human	0.6	[28]
NGF	Mouse	0.2	[21]
IFN-γ	Chicken	0.5	[29]
TNF-α	Cow	0.4	[30]
GM-CSF	Cow	0.2	[21]
GH	Flounder	0.2	[31]

## Supplementary Materials

The following are available online at https://www.mdpi.com/1660-3397/17/2/128/s1, Table S1: Bacteria and plasmids used in the study. Table S2: Medium compositions used in this study.
