# Supplementary material for: Heterologous Expression of the Marine-Derived Quorum Quenching Enzyme MomL Can Expand the Antibacterial Spectrum of Bacillus brevis
Source: Mar Drugs. 2019 Feb 21;17(2):128. doi: 10.3390/md17020128 (PMC6409708; doi:10.3390/md17020128)
Supplement: Supplementary file 1 [file marinedrugs-17-00128-s001.pdf]

Supplementary Materials

Heterologous Expression of the Marine-Derived Quorum Quenching Enzyme MomL Can Expand the Antibacterial Spectrum of *Bacillus brevis*

Jingjing Zhang [1], Jiayi Wang [1], Tao Feng [1], Rui Du [1], Xiaorong Tian [1], Yan Wang [1,2,3,*] and Xiao-Hua Zhang [1,2,3]

[1] MOE Key Laboratory of Marine Genetics and Breeding, College of Marine Life Sciences, Ocean University of China, Qingdao 266003, China; jingjingzhangnn@163.com (J.Z.); wangjiayi109911@163.com (J.W.); fengtao246@163.com (F.T.); drury612@163.com (R.D.); tianxr123@sina.com (X.T.); xhzhang@ouc.edu.cn (X.Z.)
[2] Laboratory for Marine Ecology and Environmental Science, Qingdao National Laboratory for Marine Science and Technology, Qingdao 266071, China
[3] Institute of Evolution & Marine Biodiversity, Ocean University of China, Qingdao 266003, China
* Correspondence: wangy12@ouc.edu.cn; Tel.: +86-532-8203-2721.

Supplementary Materials

Table S1. Bacteria and plasmids used in the study……………………………………………………1

Table S2. Medium compositions used in this study…………………………………………………..2

Table S1. Bacteria and plasmids used in the study.

Strains or Plasmids	Relevant Characteristics and Purpose
**Strains**	
BL21 (DE3)	Competent cell and be used as a host for protein expression
JM109	Competent cell; *recA*1, *endA*1, *hsdR*17 thi, *hsdR*17 (rk^-mk^+), *e*14⁻ (*mcrA*⁻), *supE*44, *relA*1, △(*lac*⁻*proAB*)/F'[*traD*36, *proAB*⁺, *lacIq*, *lacZ*△M15]
*Bb*MomL	A strain of *B. brevis* containing plasmid pNCMO2-*momL*
Pectobacterium carotovorum subsp. *carotovorum*	Soft rot pathogenic bacteria of plant, AHL producer
Pseudomonas aeruginosa PAO1	Ubiquitous and metabolically versatile opportunistic pathogen
Muricauda olearia Th120	Belonging to the class *Flavobacteriia* and has strong AHL degradation activity.
Chromobacterium violaceum CV026	AHL indicator strain
Agrobacterium tumefaciens A136	AHL indicator strain
**Plasmids**	
pNCMO2	The plasmid pNCMO2, the *E. coli*–*B. brevis* shuttle vector, was used as the expression vector. The P2 promoter, derived from a cell wall protein of the host bacterium, was used as the promoter for pNCMO2 expression.
pUCm-T	The pUCm-T vector is an ideal vector for cloning A-terminal PCR products. The recombinant clones with inserted fragments can be screened by blue and white spots.

Table S2. Medium compositions used in this study.

**Medium Composition**	
**2216 E liquid Medium (1 L)**	
Yeast extract	1 g
Peptone	5 g
$FePO_4$	0.1 g
Dissolved in 1000 mL seawater and adjust to pH 7.6.	
2216 E plates (1 L)	
Suspend 20 g of agar in 1000 mL of 2216 E liquid medium and sterilize 121 °C for 20 min using an autoclave. Let stand at room temperature until it has cooled to approximately 50 °C. Mix gently then dispense into plates.	
**MTNm liquid medium (1 L)**	
Glucose*[1]	10.0 g
Polypeptone	10.0 g
Meat Extract	5.0 g
yeast extract	2.0 g
$FeSO_4 \cdot 7H_2O$	10 mg
$MnSO_4 \cdot 4H_2O$	10 mg
$ZnSO_4 \cdot 7H_2O$	1 mg
$MgCl_2 \cdot 6H_2O$	4.1 g
Dissolved in 1000 mL distilled water and adjust to pH 7.0.	
**MTNm plates**	
Suspend 15 g of agar in 1000 mL of MTNm liquid medium and sterilize 121 °C for 20 min using an autoclave. Let stand at room temperature until it has cooled to approximately 50 °C and then add neomycin solution (10 mg/mL stock solution) to a final concentration of 10 μg/mL. Mix gently then dispense into plates.	
Luria-Bertani liquid medium (LB, 1 L)	
Yeast extract	5 g

Peptone	10 g
NaCl	10 g
Dissolved in 1000 mL distilled water and adjust to pH 7.0.	
**Luria-Bertani plates**	
Suspend 20 g of agar in 1000 mL of LB liquid medium and sterilize 121 °C for 20 min using an autoclave. Let stand at room temperature until it has cooled to approximately 50 °C and then add different concentrations of antibiotics. Mix gently then dispense into plates.	
***Pseudoomonas* broth (PB, 1 L)**	
Peptone	20 g
MgCl2	1.4 g
K2SO4	10 g
Glycerine	10 mL
Dissolved in 1000 mL distilled water and adjust to pH 7.6.	

*1: Sterilize glucose and glucose-free media separately. Mix after sterilization.
